# District-level health management and health system performance

**DOI:** 10.1371/journal.pone.0210624

**Published:** 2019-02-01

**Authors:** Netsanet Fetene, Maureen E. Canavan, Abraham Megentta, Erika Linnander, Annabel X. Tan, Kidest Nadew, Elizabeth H. Bradley

**Affiliations:** 1 Yale Global Health Leadership Initiative, New Haven, Connecticut, United States of America; 2 Yale School of Medicine, New Haven, Connecticut, United States of America; 3 Yale School of Public Health, New Haven, Connecticut, United States of America; 4 Vassar College, Poughkeepsie, New York, United States of America; Makerere University, UGANDA

## Abstract

Strengthening district-level management may be an important lever for improving key public health outcomes in low-income settings; however, previous studies have not established the statistical associations between better management and primary healthcare system performance in such settings. To explore this gap, we conducted a cross-sectional study of 36 rural districts and 226 health centers in Ethiopia, a country which has made ambitious investment in expanding access to primary care over the last decade. We employed quantitative measure of management capacity at both the district health office and health center levels and used multiple regression models, accounting for clustering of health centers within districts, to estimate the statistical association between management capacity and a key performance indicator (KPI) summary score based on antenatal care coverage, contraception use, skilled birth attendance, infant immunization, and availability of essential medications. In districts with above median district management capacity, health center management capacity was strongly associated (p < 0.05) with KPI performance. In districts with below median management capacity, health center management capacity was not associated with KPI performance. Having more staff at the district health office was also associated with better KPI performance (p < 0.05) but only in districts with above median management capacity. The results suggest that district-level management may provide an opportunity for improving health system performance in low-income country settings.

## Introduction

Developing management and problem-solving capacity at all levels is fundamental to health systems strengthening. Competencies of health management include strategic thinking and problem solving, human resource management, financial management, operations management, performance management and accountability, governance and leadership, political analysis and dialogue, and customer and community assessment and engagement [1]. Effective management is particularly critical in low-income settings where scarce resources must be carefully stewarded and efficiently deployed to meet the substantial health needs of the population. To date, much of the literature on improving health management has been conducted at the level of health centers or hospitals and has largely demonstrated that practical education and mentoring in management can promote significant improvements in the quality and consistency of health service delivery [2–12]. These studies have tracked a variety of health service measures such as waiting times [2, 5], pharmacy stock outs [4, 6], human resource management [6, 7], infection control processes [6], medical record availability [4, 9], and information system implementation and data monitoring [10–12].

Despite the plethora of studies examining facility-level (e.g., health center and hospital) management capacity and health service delivery outcomes, we could find only a handful of studies that assessed the relationship between district-level health management and health system performance. Three case studies [3, 13, 14] used the Challenge Model as part of the Leadership Development Program (LDP) developed by Management Sciences for Health (MSH) in Ghana, Egypt, and Mozambique, respectively. In these case studies, the LDP intervention was linked to improved skilled birth attendance and fewer still births in Ghana [3], improved antenatal and postnatal care visits and reductions in maternal mortality in Egypt [13], and increased percentage of attended births in Mozambique [14]. An additional pre-post interventional study [15] indicated a positive association between the LDP intervention and improved antenatal care visits, skilled birth attendant deliveries, and fully-immunized children in Kenya. Last, using a mixed methods approach, researchers documented increased antenatal care coverage and skilled birth attendant rates attributed in part to increased district management capacity as part of the Ethiopian Millennium Rural Initiative [8].

While these studies are helpful, the literature examining the influence of district-level management on population outcomes remains limited. First, few studies exist. Second, most are case studies, limiting their generalizability. Third, most commonly, the studies use exposure to the LDP model as the indicator of management capacity rather than employing a quantifiable and more precise measure of management capacity. Fourth, the studies lack statistical analysis to establish the significance of the relationships between management capacity and population-based outcomes. Last, none of the studies examines the impact of improved district-level management in conjunction with health center-level management. As a result, the association between management capacity at the district health office level and how it may interact with management capacity at the health center level to produce population-level health outcomes remains largely unknown.

Accordingly, we sought to examine the variation in district level health management capacity and examine its association with health system performance including contraception use, antenatal care, skilled birth attendance, immunization rates, essential drug availability. We also examined the potentially interaction effects of district-level and health center-level management capacity in their association with population-based health outcomes. We chose to pursue this inquiry in Ethiopia where the Ministry of Health has endorsed a national plan to enhance district health office and health center capacity as key to a robust primary care system. Findings from this study can be useful in understanding the potential leverage of improving management capacity, as well as the comparative importance of targeting management capacity building efforts at district health offices, health centers, or both.

## Methods

### Study design and sample

We conducted a cross-sectional analysis of data collected during the baseline assessment for a 3-year interventional study to improve targeted district-level health outcomes in Ethiopia, the Primary Healthcare Transformative Initiative (PTI). PTI is led by the Federal Ministry of Health with support from Yale University and The Bill & Melinda Gates Foundation to improve management capacity and performance within district health offices. Districts are called woredas in Ethiopia, and are the third level administrative division of the country, following regions and zones. Woreda health offices oversee and coordinate primary care services for catchment areas of approximately 200,000 population, including oversight of 4–5 health centers, 20–30 health extension workers, and, in some cases, a primary hospital. In collaboration with the Federal Ministry of Health and Regional Health Bureaus, 36 woredas from 4 regions were selected as PTI sites (9 in Amhara, 15 in Oromia, 9 in SNNPR, and 3 in Tigray). These woredas include 226 health centers covering a catchment area of more than 5.5 million population. Five health centers (3 in Amhara and 2 in Tigray) were dropped from the statistical analyses due to missing data (97.8% health center inclusion rate). Formal institutional board review approval was deemed unnecessary by the Yale Human Subjects Committee because we collected health center and district-level data in aggregate. No individual-level health information was obtained.

### Data collection

Our analysis used data collected as part of the baseline measures for PTI, to examine the association between management capacity at the woreda health office and health center levels and health system performance at the start of PTI. In each woreda and health center, data were collected by PTI management mentors, who were assigned to each woreda and trained to promote data reliability and quality assurance, for the quarter spanning October to December 2015 from the district health office directors and their delegates, including health center directors, health center department heads, and other relevant woreda and health center staff. The data collection process included review of relevant official documents and records, and direct observations of the availability of the required standards or services across the woreda health offices and all health centers within study woredas.

### Dependent variables

The dependent variable was a key performance indicator (KPI) summary score calculated from the performance on 5 indicators, which were prioritized by the Federal Ministry of Health and Regional Health Bureaus, a subset of the 18 KPIs prioritized by the Federal Ministry of Health and Regional Health Bureaus as part of the Health Services, Development, and Planning (HSDP) national planning efforts because they were most consistently reported with reliable data quality [16]. The five indicators were: 1) antenatal care coverage (ANC), i.e. the number of women having 4 or more antenatal care visits divided by the number of expected births in the health center catchment area; 2) contraceptive acceptance rate (CAR), i.e., or the number of women reporting the use of modern contraception divided by the estimated number of women in child bearing years who are not pregnant in the health center catchment area; 3) skilled birth attendance rate (SBA), i.e., the number of women who give birth in a health facility divided by expected number of births in the health center catchment area; 4) the percent of 1-year old children who have received all recommended immunizations in the health center catchment arear; and 5) essential drug availability, i.e., the average percentage of 22 essential drugs to be found in stock per month at health centers. Performance on each of the 5 indicators, reported as a percentage from 0–100%, was normally distributed. To create a summary KPI score for each health center, performance on the 5 indicators was averaged to create a KPI summary score that could range from 0–100% [17]. We had no apriori reason to weight the 5 indicators. Whether the five indicators were summed or averaged did not affect the results, and we found averaging to be the most intuitive for policy makers and practitioners.

### Independent variables

Our primary independent variable was management capacity, measured both at the woreda health office and health center levels. At the woreda health office level, management capacity was ascertained using adherence to the woreda management standards (WMS), which is a regionally endorsed composite of 26 standards organized in 5 domains: governance and organizational capacity, service delivery, collaboration with other sectors, community engagement, and performance management (S1 and S2 Tables). For each woreda health office, adherence to WMS was calculated as the percent of the 26 standards that were fully met. Management capacity at the health center level was measured using the Ethiopia Health Center Reform Implementation Guidelines (EHCRIG, October 2015, current at time of study) (S3 Table), a nationally endorsed set of 99 standards for health center management in 11 domains: leadership and governance, health post support, patient flow, medical records management, pharmacy services, laboratory services, infection prevention safety, medical equipment management, human resource management, performance quality improvement, and financial management. For each health center, adherence to EHCRIG was measured as the percent of the 99 standards that were met. Last, we included variables representing the number of staff employed within the woreda health office and the regional location (Amhara, Oromia, Tigray, or SNNP).

### Statistical analysis

We used standard descriptive statistics to characterize health centers and woredas in terms of region, staffing, management capacity at health centers (measured by EHCRIG) and at woreda health offices (measured by WMS), and the KPI summary score. We estimated the unadjusted association between the KPI summary score and management capacity at the health center and woreda health office, total staffing in the woreda, health center catchment area population, and region. We used generalized linear regression models that accounted for the cluster design of sampling of health centers clustered within woredas within regions. We used survey regression analysis with a cluster statement to ensure that the standard deviation of the regression coefficients was correctly estimated to account for the distribution of health centers within woredas. Because the measures of management capacity at the health center level and at the woreda health office level were strongly correlated (Pearson correlation coefficient: 0.56, p < 0.001), we tested a model with the interaction of these variables and found the interaction to be statistically significant (p = 0.03). As a result, we reported adjusted associations between the KPI summary score and independent variables overall and stratified by above and below median woreda management capacity. All analyses were conducted accounting for the clustering of health centers within a woreda. We employed p < 0.05 as the threshold for statistical significance for all analyses. We used SAS software, version 9.2 (SAS institute, Cary, NC) for statistical analysis. All underlying deidentified data are available from the Dryad repository at doi:10.5061/dryad.gc974r7.

## Results

### Description of health centers and woredas

Our sample included 221 health centers, representing 97.8% of all health centers approached. A total of 33.5% (n = 74) of the health centers were located in Amhara; 37.6% (n = 83) in Oromia, 22.6% (n = 50) in SNNPR, and 6.3% (n = 14) in Tigray region. Of the 36 woredas, 25.0% (n = 9) were located in Amhara, 41.7% (n = 15) in Oromia, 25.0% (n = 9) in SNNPR, and 8.3% (n = 3) in Tigray. The number of staff in the woreda health office averaged 23.3 with SD of 4.7.

KPI summary scores varied (Table 1), with a mean of 66% and standard deviation (SD) of 21.2 percentage points. The mean across the health centers for ANC coverage was 64% (SD 28.1 percentage points); mean CAR was 58% (SD 30.1 percentage points); the mean SBA rate was 64% (SD 28.2 percentage points), the mean percent of 1-year olds fully immunized was 71% (SD 29.8 percentage points), and the mean percent of essential drugs available was 73 (SD 29.6 percentage points). The health center EHCRIG scores also varied, with mean 41%, median 39%, and SD 15.2. The woreda WMS score also varied across woredas, with mean 43%, median 42%, and SD 15.1 percentage points.

**Table 1:** Mean and standard deviation (SD) of health center and woreda characteristics among Ethiopian health centers (N = 221).

	Overall	Amhara	Oromia	SNNP	Tigray
Characteristic	n = 221	n = 74	n = 83	n = 50	n = 14
**KPI summary score**	66.0% (28.1)	66.1 (18.2)	70.8 (19.5)	63.4 (24.5)	45.8 (20.9)
**Individual indicators**					
ANC^1^ rate	63.6% (28.1)	65.8% (26.1)	70.1% (26.1)	53.1% (30.9)	50.9% (27.0)
CAR^2^	58.4% (30.2)	57.2% (32.1)	63.4% (26.9)	56.5% (32.5)	43.0% (22.8)
SBA^3^ rate	63.6% (28.2)	61.5% (25.7)	70.9% (24.8)	53.1% (33.5)	73.4% (26.6)
Full immunization coverage	71.4% (29.8)	71.6% (27.1)	75.3% (29.5)	64.7% (32.6)	76.6% (34.7)
Essential drug availability	73.4% 29.6)	73.5% (30.7)	74.6% (24.2)	88.0% (7.7)	6.2% (11.2)
**Health center management (EHCRIG**^4^**)**	41.3 (15.2)	38.8 (15.1)	39.7 (13.6)	43.8 (17.4)	48.1 (13.5)
**Woreda health office management (WMS**^5^**)**	41.5 (15.2)	42.2 (11.5)	39.4 (13.9)	49.0 (19.4)	54.1 (14.1)

^1^ ANC: Number of women reporting the use of modern contraception divided by the estimated number of women in child bearing years

^2^ CAR: Number of women reporting the use of modern contraception divided by the estimated number of women in child birth years

^3^ SBA: Number of women who give birth in health facility divided by expected number of births

^4^ EHCRIG: Ethiopia Health Center Reform Implementation Guidelines

^5^ WMS: Woreda Management Standards (N = 36 woredas)

### Association between management and key performance outcomes

In unadjusted analysis, health centers with above median management capacity had significantly better performance on the KPI summary score (KPI scores 73% versus 60%, respectively, or 13 percentage point difference; p <0.01) (Table 2). Health centers located in woredas with above median management capacity also had higher KPI summary scores (KPI scores 70% versus 62%, or 8 percentage point difference), but this difference was not statistically significant (p = 0.15). Neither region nor level of staffing in the woreda were significantly associated with KPI performance (p > 0.05).

**Table 2:** Unadjusted associations with KPI summary score (N = 221 health centers).

Independent variable	Change in KPI summary score (percentage points)	p^3^
**Total number of staff in woreda health office**	0.70	0.35
**Region**		
Amhara	**Reference**	
Oromia	4.63	0.43
SNNPR	-2.75	0.77
Tigray	-20.29	0.06
**Management capacity at health center (EHCRIG**^1^ **score)**	13.20	<0.01
**Management capacity at woreda health office (WMS**^2^ **score)**	8.42	0.15

^1^ EHCRIG: Ethiopia Health Center Reform Implementation Guidelines

^2^ WMS: Woreda Management Standard (17 woredas scored below the median WMS score; 19 scored above the median)

^3^Statistical test: generalized linear regression model with robust standard errors adjusted for clustered design at the woreda level

In adjusted models, the associations between health center management capacity and KPI summary scores differed significantly by woreda management capacity, as shown in stratified analysis (Table 3). Among woredas with above median management capacity (measured by WMS), health center management capacity (measured by EHCRIG) was significantly associated with better KPI performance (p = 0.03) whereas this association was not significant in woredas with low management capacity (p = 0.96). Higher staffing levels were also associated with better KPI performance but only in woredas with above median management capacity (p = 0.01); regional differences were pronounced, particularly among woredas with poor management capacity.

**Table 3:** Adjusted associations of health centers with KPI summary score, overall and stratified analysis.

	Change in KPI summary score (percentage points)					
Independent variable	Total Sample N = 221		Among woredas with below median WMS^2^ score N = 112		Among woredas with above median WMS^2^ score N = 109	
	Change	p^3^	Change	p^3^	Change	p^3^
**Total number of staff in woreda health office**	0.17	0.84	-0.62	0.63	1.82	0.01
**Region**						
Amhara	*Reference*	--	*Reference*	--	*Reference*	--
Oromia	2.49	0.73	14.80	0.05	-19.14	0.02
SNNPR	-4.62	0.59	-8.95	0.55	-7.68	0.35
Tigray	-25.12	0.02	-40.98	<0.01	-12.23	0.28
**Management capacity at health center (EHCRIG**^1^ **score)**	12.88	0.01	-0.22	0.96	17.26	0.03
**Management capacity at woreda health office (WMS**^2^**score)**	5.49	0.34	n/a	n/a	n/a	n/a
**Adjusted r**^2^	0.32		0.30		0.46	

^1^ EHCRIG: Ethiopia Health Center Reform Implementation Guidelines

^2^ WMS: Woreda Management Standard (17 woredas scored below the median WMS score; 19 scored above the median)

^3^Statistical test: generalized linear regression model with robust standard errors adjusted for clustered design at the woreda level

## Discussion

In this study, we found that woredas with better management capacity at both the woreda health office and the health center levels achieved significantly better performance in a summary score of key performance indicators including ANC, CAR, SBA, immunization rates, and availability of essential medicines. Importantly, the management capacity at the woreda health office level modified the effect of management capacity on KPI performance at the health center level; we found that having stronger management at the woreda health office magnified the positive effects of strong management at the health center level. We also found that having more staff employed at the woreda health office was associated with better KPI performance but only among those woredas with stronger management capacity. In both cases, this likely because added staffing, coordination, and accountability structures—all fundamental to management capacity—may prompt greater productivity, collaboration, and focus on performance than in woredas with lower management capacity. Previous work in low-income countries has suggested the importance of management on the functioning of health centers and hospitals [2–12], but fewer have demonstrated the link between district-level management and health indicators in the population the district health office serves.

Policymakers have called for greater attention on the district-level as part of health system strengthening, but much of this work has focused on district-level planning and data monitoring. We have argued that management skills–such as strategic problem solving, human resource management, financial management, and operations management–are fundamental to health system strengthening [1, 18]. In contrast to a health facility, which delivers healthcare services, the district level health office focuses on enabling facilities to deliver services through regulatory, monitoring, and oversight functions. Thus, management skills at the woreda health office—including governance, performance data review, collaboration with non-health sectors, community engagement, and performance management—are essential to planning, ensuring resources are available, and holding staff accountable to provide and monitor services. Furthermore, this study demonstrates management capacity can be feasibly measured with quantitative data, providing a potentially powerful lever to target for improvement as part of health system strengthening efforts in low-income settings.

The results of the study should be interpreted in light of its limitations. First, this was a cross-sectional study demonstrating a statistical association between management capacity and primary health system performance. We are unable to establish causality from this observation study; however, these early results suggest that longitudinal studies to examine changes over time are warranted. Second, the sample size is relatively limited and the study was conducted in a single country. Although we were able to detect statistically significant differences in this sample, larger studies with more statistical power as well as greater generalizability would be helpful. Third, we were unable to examine differences in key performance indicators based on the sub-components of management capacity. Future larger studies with greater variation across sub-components may reveal such patterns. Notably, longitudinal evaluation of changes in management practices and performance is across Ethiopia is ongoing and may allow such analysis. Fourth, we had limited data on sociodemographic and economic factors across woredas. Although we knew the region of the woreda, we did not have data in its wealth or education, both of which may be important influences on key performance indicators. Last, the quality of health data in low-income countries has been shown to be limited [19, 20]. To address data quality issues, we used explicit protocols and conducted substantial training and supervision of data collectors. Nevertheless, we anticipate that the misclassification bias arising from errors in data collection are likely non-differential, thus leading to a conservative bias toward the null. Advances underway in Ethiopia in public health data collection and reporting will likely improve the reliability of future such studies.

In summary, this is one of the first studies of which we know to examine the association between a detailed measures of management capacity at the district level and outcomes in the population served by that district. We find that although the management at the health center level is critical, its effect is strengthened by management capacity at the district health office level. The work highlights a key, often neglected leverage point in health system strengthening: management capacity. Policymakers might focus greater attention on the development of management capacity, particularly at the district level; management capacity can be reliably measured and can an additional input with which to achieve better district-level coverage and health facility readiness. The work in Ethiopia provides encouraging evidence supporting the feasibility of measuring and reporting on management capacity and its potential to promote public health outcomes in resource limited settings.

## Supporting information

S1 Table Woreda management standards. (PDF)

S2 Table Woreda management standards (detailed). (PDF)

S3 Table Ethiopian Health Center Reform Implementation Guidelines (EHCRIG). (PDF)
