# Supplementary material for: District-level health management and health system performance
Source: PLoS One. 2019 Feb 1;14(2):e0210624. doi: 10.1371/journal.pone.0210624 (PMC6358064; doi:10.1371/journal.pone.0210624)
Supplement: S1 Table — (PDF) [file pone.0210624.s001.pdf]

S1. Woreda Management Standards

**Chapter 1: Governance and Organizational Capacity**
1. The organizational structure of the WoHO reflects its core functions. The organizational structure of the WoHO has core processes and case teams responsible to execute the following core functions.
2. WoHO ensures governing boards of Primary Health Care Facilities (HCs, PHs) are functional.
3. Coordination and communication among governing boards of Primary Health Care Facilities.
4. WoHO and Primary Health Care Facilities is led by qualified personnel
5. WoHO ensures Primary Health Care Facilities are staffed.
6. WoHO ensure adequate finance allocation and provides financial oversight to Primary Health Care Facilities (Budget vs actual reports compiled by the WoHO)
7. WoHO provides oversight and facilitates procurement of goods and services by Primary Health Care Facilities.
8. WoHO ensures that Primary Health Care Facilities have basic infrastructure requirements: buildings, communications, electricity, water.
**Chapter 2: Service Delivery**
9. WoHO ensures availability of essential package of basic health care services at Primary Health Care Facilities.
10. There is a referral and linkage system between Primary Health Care Facilities in the Woreda.
11. WoHO coordinates quarterly clinical audits in all Primary Health Care Facilities to ascertain adherence to clinical guidelines, SOPs.
12. WoHO coordinates clinical mentoring between primary hospital and health centers.
13. Monitor outbreak and public health emergencies (Surveillance report and Emergency response plan)
**Chapter 3: Community Engagement**
14. Community are organized in 1-5 networks and developments teams (Health Development group command post reports).
15. All kebeles in the Woreda are verified as model in health service delivery.
16. Establish and maintain community feedback mechanisms (Town hall meeting minutes and Community score card report).
17. Woreda starts and maintains Community Based Health Insurance (CBHI) scheme.
18. WoHO coordinates community contribution and ownership on community based public health interventions.
**Chapter 4: Coordination with other key sectors in the Woreda**
19. Inter-sectoral coordination mechanisms established.
20. Coordinate and align activities of development partners, and civil society organizations.
21. Private health facilities work in alignment with priorities of the Woreda and operate within the national regulatory framework.
**Chapter 5: Performance Management**
22. WoHO develops Woreda based plan and targets.
23. System for performance review established and operational in Primary Health Care Facilities.

24. Performance of Primary Health Care Facilities is monitored using evidence from; KPIs, EHRIG, EHCRIG.
25. WoHO compiles and disseminates national and regional policies, guidelines and manuals used as references for performance management.
26. Supportive supervisions to Primary Health Care Facilities (Supportive supervision report)
