# Supplementary material for: District-level health management and health system performance
Source: PLoS One. 2019 Feb 1;14(2):e0210624. doi: 10.1371/journal.pone.0210624 (PMC6358064; doi:10.1371/journal.pone.0210624)
Supplement: S2 Table — (PDF) [file pone.0210624.s002.pdf]

S2. Woreda Management Standards (Detailed)

Governance and Organizational Capacity	
**WMS1**	**The organizational structure of the WoHO reflects its core functions. The organizational structure of the WoHO has core processes and case teams responsible to execute the following core functions.**
	a. Planning, monitoring and evaluation of health promotion, disease prevention and curative health care activities in the Woreda.
	b. Coordinating mentoring and technical support among Primary Health Care Facilities, (Primary Hospital, Health Centers and Health Posts)
	c. Planning and coordinating supportive supervision of Primary Health Care Facilities, and monitor quality of service.
	d. Coordinate resource mobilization for primary health care.
	e. Ensuring community engagement and ownership
	f. Disease surveillance and coordinating and planning. emergency response for public health emergencies.
	g. Conducting regulatory functions.
	h. Coordination with other sectors at the Woreda level.
	i. Provision of oversight on finance, human resources infrastructure and supplies to Primary Health Care Facilities.
**WMS 2**	**WoHO ensures governing boards of Primary Health Care Facilities (HCs, PHs) are functional.**
	a. All governing boards meet monthly. WoHO, presenting the case to the Woreda administration, ensures that corrective action is taken on governing boards that do not meet monthly.
	b. Minutes of governing board meetings include; review of action points from previous meeting, performance review using KPIs and way forward action points.
	c. Financial and programmatic performance targets are reviewed using facility-level Key Performance Indicators (KPIs), quarterly.
**WMS 3**	**Coordination and communication among governing boards of Primary Health Care Facilities.**
	a. Woreda Administrator organizes quarterly joint meeting of all governing board representatives from Primary Hospitals (PHs) and Health Centers (HCs).
	b. Minutes of governing board joint meetings include; review of action points from previous meeting, performance review using KPIs and way forward action points.
	c. Priorities of governing boards are defined, financial and programmatic performance targets are reviewed, using KPIs combined from Primary Health Care Facilities.
**WMS 4**	**WoHO and Primary Health Care Facilities is led by qualified personnel**
	a. WoHO head has educational qualification and experience based on requirements of Ethiopian civil service guidelines.
	b. PHCU Directors have educational qualification and experience based on requirements of Ethiopian civil service guidelines.
	c. CEOs of primary hospitals have educational qualification and experience based on requirements of Ethiopian civil service guidelines.

	d. All primary health care facility and Woreda managers have certificate-based, on-the-job management, leadership, problem solving training.
**WMS 5**	**WoHO ensures Primary Health Care Facilities are staffed.**
	a. WoHO keeps updated record of staffing of; WoHO and Primary Health Care Facilities (filled positions and vacancies)
	b. WoHO ensures that WoHO and Primary Health Care Facilities are staffed with the required number and qualification of staff.
	c. WoHO ensures equitable allocation (professional mix and number) of health professionals among the Primary Health Care Facilities in the Woreda based on patient volume.
	d. WoHO recognizes and motivates high performing WoHO and primary health care facility staff.
**WMS 6**	**WoHO ensure adequate finance allocation and provides financial oversight to Primary Health Care Facilities (Budget vs actual reports compiled by the WoHO)**
	a. WoHO secures a minimum of 15% budget allocation to health from the total Woreda level government expenditure.
	b. WoHO reviews income statements and balance sheets, and provides feedback to all Primary Health Care Facilities quarterly.
	c. WoHO monitors execution of internal financial audits of Primary Health Care Facilities every six months.
	d. WoHO in coordination with Woreda finance plans and executes external financial audits for all Primary Health Care Facilities annually.
**WMS 7**	**WoHO provides oversight and facilitates procurement of goods and services by Primary Health Care Facilities.**
	a. WoHO compiles procurement needs (goods and services) including essential drugs by each primary hospital and health centers.
	b. WoHO monitors that procurement are executed timely by primary hospital and health center and maintains checklist showing list of items requested vs procured.
	c. WoHO has ensured implementation of LMIS (Logistics Management Information System) in Primary Health Care Facilities.
	d. The WoHO oversees zero stock out rates across all essential drugs Primary Health Care Facilities.
**WMS 8**	**WoHO ensures that Primary Health Care Facilities have basic infrastructure requirements: buildings, communications, electricity and water.**
	a. WoHO keeps a record of status of buildings, communications, electricity, and water availability for each primary health care facility.
	b. WoHO develops a plan of action in consultation with facility governing boards, Woreda Administration and other relevant stakeholders to fill identified infrastructure gaps in building, communications, electricity, and water.

2. Service Delivery	
**WMS 9**	**WoHO ensures availability of essential package of basic health care services at Primary Health Care Facilities.**
	a. WoHO maintains an updated record of essential package of basic health care services available in each Primary Health Care Facilities.
	b. WoHO ensures essential package of basic health care services are available in each Primary Health Care Facilities.
**WMS 10**	**There is a referral and linkage system between Primary Health Care Facilities in the Woreda.**
	a. All Primary Health Care Facilities use standard referral protocol including standard referral forms and registers.
	b. WoHO organizes referral feedback meeting quarterly between Primary Health Care Facilities where data on referral, feedback, and unnecessary referrals are reviewed.
	c. Ambulance administration policy is in place and operational.
**WMS 11**	**WoHO coordinates quarterly clinical audits in all Primary Health Care Facilities to ascertain adherence to clinical guidelines, SOPs.**
	a. WoHO identifies clinical audit areas for each primary health care facility in collaboration with Primary Health Care Facilities.
	b. WoHO monitors Primary Health Care Facilities conduct clinical audits on the areas identified quarterly.
	c. WoHO ensures implementation of quality improvement activities in all Primary Health Care facilities based on clinical audit findings.
**WMS 12**	**WoHO coordinates clinical mentoring between primary hospital and health centers.**
	a. WoHO working with the CEO of Primary Hospital organizes clinical mentoring sessions between primary hospital and all health centers monthly. (Clinical mentoring may include one-on-one case management and observation, chart reviews, attachments, and didactic sessions).
	b. WoHO organizes knowledge and skills assessments of mentees semi-annually in collaboration with primary hospital to monitor the outcome of mentoring.
	c. WoHO organizes trainings or mentoring sessions in collaboration with the primary hospital based on knowledge and skills gap identified.
**WMS 13**	**Monitor outbreak and public health emergencies (Surveillance report and Emergency response plan)**
	a. Case surveillance for reportable diseases is in place.
	b. Resources and systems to respond to public health emergencies are in place.

3. Community Engagement	
**WMS 14**	**Community are organized in 1-5 networks and developments teams (Health Development group command post reports).**
	a. All health development teams and 1-5 network leaders have undergone training and started implementation of activities.
	b. All health development teams are functional (meet regularly and document minutes of meetings).
	c. All health development team leaders are accredited to level 1 qualification.
**WMS 15**	**All kebeles in the Woreda are verified as model in health service delivery.**
	a. WoHO compiled and analyses health extension package performance of all kebeles and categorizes them into; high, middle and low performers.
	b. Woreda organizes annual mobilization (ignition) meeting with the community focusing on creation of model kebeles.
	c. WoHO monitors model kebele initiative performance quarterly.
**WMS 16**	**Establish and maintain community feedback mechanisms (Town hall meeting minutes and Community score card report).**
	a. WoHO working closely with the Woreda administration, Health Centers and Primary Hospitals organizes quarterly community town hall meetings where community provides feedback on quality and access to services.
	b. A system of community score card is established and maintained at Primary Health Care Facilities quarterly.
	c. WoHO in consultation with Primary Health Care Facilities, coordinates the implementation of activities responding to feedback from the community quarterly.
**WMS 17**	**Woreda starts and maintains Community Based Health Insurance (CBHI) scheme.**
	a. The Woreda meets and maintains minimum community enrollment to start CBHI.
	b. WoHO in collaboration with Woreda administration organizes quarterly meetings with CBHI agency, PHC facilities, and kebele administration to review progress on CBHI.
**WMS 18**	**WoHO coordinates community contribution and ownership on community based public health interventions.**
	a. WoHO has identified health projects which can be implemented with monitory and in-kind support from the community.
	b. WoHO coordinates monetary and in-kind community contributions in infrastructure projects such as building health posts, HEW residences, community latrines, maternity waiting areas, and other projects.
	c. WoHO oversees and coordinates all monetary and in-kind contributions of the community and reports back to community on achievements.

4. Coordination with other key sectors in the Woreda	
**WMS 19**	**Inter-sectoral coordination mechanisms established.**
	a. WoHO ensures integration of health sector related activities with other sectors' plans such as education, agriculture, infrastructure, electricity, water, finance, civil service, and others.
	b. WoHO implemented joint planned activities with other sectors.
	c. Woreda level inter-sectoral steering committees monitor jointly planned activities and makes decisions to address potential bottlenecks quarterly.
**WMS 20**	**Coordinate and align activities of development partners, and civil society organizations.**
	a. WoHO maintains updated mapping of development partners, and civil society organizations working in the health sector.
	b. WoHO works with the Woreda administration to organize joint planning and review meetings with development partners, and civil society organizations working in the health sector quarterly.
**WMS 21**	**Private health facilities work in alignment with priorities of the Woreda and operate within the national regulatory framework.**
	a. WoHO has included all private facilities related with the health sector in the Woreda such as; private health facilities, food and drink provides, schools, industrial sites, etc. in its regulatory plan.
	b. WoHO organizes periodic inspections in private facilities based on FMHACA regulatory standards and set up a system for follow-up.
	c. WoHO organizes joint consultations with private facilities semi-annually.

5. Performance Management	
**WMS 22**	**WoHO develops Woreda based plan and targets.**
	a. WoHO develops 5-year strategic plan for health.
	b. WoHO develops Woreda based annual plan and targets aligned with strategic plan and allocates targets to Primary Health Care Facilities.
	c. WoHO organizes quarterly review meetings with the participation of Primary Health Care Facilities and stakeholders to review implementation of activities and provide feedback.
**WMS 23**	**System for performance review established and operational in Primary Health Care Facilities.**
	a. WoHO and all Primary Health Care Facilities staff conduct monthly performance reviews based in balanced score card and semi-annually 360 performance evaluations.
	b. WoHO monitors implementation of result oriented performance review in Primary Health Care Facilities semi-annually and ensure performance review results are used for human resource and management decisions.
**WMS 24**	**Performance of Primary Health Care Facilities is monitored using evidence from; KPIs, EHRIG, EHCRIG.**
	a. WoHO and primary health facility managers and boards use KPIs to monitor performance and take corrective action on a monthly basis.
	b. WoHO makes quarterly EHRIG, EHCRIG assessments of PH and HCs.
	c. WoHO monitors HMIS data including; timeless, quality, and completeness of data in each of the Primary Health Care Facilities and provide feedback on a quarterly basis (LQAS).
**WMS 25**	**WoHO compiles and disseminates national and regional policies, guidelines and manuals used as references for performance management.**
	a. WoHO compiles and disseminate national and regional policies, guidelines, and manuals to all Primary Health Care Facilities.
	b. WoHO monitors availability of a list of policies, guidelines and manuals at Primary Health Care Facilities quarterly.
**WMS 26**	**Supportive supervisions to Primary Health Care Facilities (Supportive supervision report)**
	a. WoHO uses standardized integrated supportive supervision checklist customized to each level of Primary Health Care Facilities and community level.
	b. WoHO conducts integrated supportive supervision quarterly, jointly with relevant stakeholders, and written feedback is given to all Primary Health Care Facilities in not more than 2 weeks after the visit.
	c. WoHO puts in place a system for tracking action on feedback provided through supportive supervisions.
