# Supplementary material for: District-level health management and health system performance
Source: PLoS One. 2019 Feb 1;14(2):e0210624. doi: 10.1371/journal.pone.0210624 (PMC6358064; doi:10.1371/journal.pone.0210624)
Supplement: S3 Table — (PDF) [file pone.0210624.s003.pdf]

S3. Ethiopian Health Center Reform Implementation Guidelines (EHCRIG)

Spring 2016

**CHAPTER 1. HEALTH CENTER LEADERSHIP AND GOVERNANCE**
1. The Health Center Governing Board is established
2. The Health Center Governing Board conducts regular meeting at least once in three months
3. The Body approves annual and strategic plans for the health center to achieve its goal of improving its community's health and welfare and evaluate its achievement versus plan every quarter.
4. The health center director is assigned by the city administration or WoHO. The health center director organizes and lead the heath center management committee comprised of different case teams.
5. The health center director is evaluated quarterly, consistent with operational and strategic plans as established by the Body and the manager collectively.
6. Health center director plan versus achievement report is monthly submitted to the WoHO
7. Free and fee for health services that clients pay at reception and cashier are posted at health notice board in clear and visible manner
8. The health center has signed memorandum of understanding agreement with entities that it provides free or credit health care services
9. The health center finance officer presents the detailed status of health center financial reports to the health center management committee
10. The health center has procurement plan approved by the health center governing body
11. Health center financial manual is approved by the health center governing board and bears the seal of the of health center on it
12. The health center finance is at least annually audited by the worked finance office
**CHAPTER 2. HEALTH POST SUPPORT AND COMMUNITY HEALTH**
13. The health center has established the necessary structural arrangement to implement linkage with health posts including assigning personal to manage and follow the system.
14. A one to five linkage is established in the catchment community and the functionality is assured
15. The health center creates linkage with 5 health posts under its custody and ensures its functionality
16. The health center prepares core plans as a PHCU for the health posts under its custody and engage the community and relevant stallholders
17. A detailed time table and plan is developed for each professional in the health center to support health extension workers at each Development Group.
18. The health center collect and analyze weekly performance reports from health posts and organize a regular review meetings to follow the overall progress in major community activities, to identify best practices and to share it among health posts.
19. The health center sends a timely and appropriate feedback to the health posts and follows the implementation of the feedback during regular support.
20. The health center organizes an integrated and a regular supportive supervision to identify major gaps and to support accordingly.
21. The health center allocates appropriate supplies to health posts and follows their appropriate use.
**CHAPTER 3. PATIENT FLOW**
22. Procedures are established to ensure efficient patient flow; such procedures are specific to emergency, outpatient and delivery services that seek to reduce patient crowding.
23. All health center staff are aware on the procedures guidelines and implement accordingly
24. The Health center has a Triage, staffed with appropriately trained personnel and equipped with necessary equipment and supplies.

25. Outpatient/client appointment systems are in place for all disciplines provided by the health center.	
26. The Health center has a Liaison and Referral Service guideline that the health facility staff understand and implement	
27. There are sign posts at the compound of the health that directs clients/patients to the desired health service units	
28. The health center has established maternity waiting room fulfilled with essential utilities such as latrine, bathroom, electric power and water	
**CHAPTER 4. MEDICAL RECORDS MANAGEMENT**	
29. The Health Center utilizes a single, unified registration system for all patients/clients, including out-patients and emergency admissions (if relevant, inpatient) or specialty clinics and also utilizes a Master Patient Index with a single, unique Medical Record Number for each patient.	
30. The Health Center utilizes a paper-based or computer-based system to track where the medical record is located at all times and also uses a standardized and uniform set of forms prepared by the FMOH or RHB to document a complete medical record for patient's/client's care.	
31. The Health Center has medical records management guidelines for proper handling and confidentiality of medical records	
32. The Health Center has orientation and training programs for all medical records personnel to ensure awareness and competency in medical record management procedures	
**CHAPTER 5. PHARMACY SERVICES**	
33. The Health Center has a Drug and Therapeutics Committee (DTC) which implements various measures designed to promote the rational, safe and cost-effective use of medicines.	
34. The Health Center has a separate pharmacy department comprising dispensaries and medical store directed by a registered Pharmacist and Pharmacist/Pharmacy technician respectively.	
35. The Health Center has a health facility specific List of Medicines classified by VEN that contains all Drugs, Medical Supplies, consumable-Medical equipment's and Reagents. The List shall be reviewed and updated annually.	
36. The health center has an effective supply chain system for pharmaceuticals, medical equipment and supplies	
37. The health center provides clear and correct prescribed drug information to patients and keep the proper record of the service	
38. The Health Center provides access to drug information to both health care providers and patients in order to optimize drug use.	
39. The Health Center has policies and procedures for identifying and managing drug use problems, including: Identifying and reporting adverse drug reactions, and prescription monitoring	
40. The Health Center has a supply and inventory management system for drugs, medical supplies and consumable equipment approved by the DTC	
41. The Health Center conducts a physical inventory of all pharmaceuticals in the store and each dispensing unit at a minimum once a year.	
42. The Health Center ensures proper and safe disposal of pharmaceutical wastes and expired drugs in line with national guidance.	
43. The health centers pharmacy assists and monitors pharmaceutical management activities at the health posts.	
44. All Units of the pharmacy service have adequate personnel, equipment, premises and facilities required to store drugs, medical supplies and equipment and carry out dispensing, and counselling services.	
45. The health center conducts internal audit twice and external audit once every year on all pharmaceuticals, equipment and supplies	
**CHAPTER 6. LABORATORY SERVICES**	
46. Current list of laboratory tests provided by the facility with the price of each test is accessible to all clinical staff and patients.	
47. Does the Laboratory management meet the needs and requirements of customers making the laboratory results are discussed up on and interpreted correctly	
48. Does the Laboratory have adequate number of staff, necessary space, and materials for work?	

49. The laboratory has a logistic management system to monitor the procurement and use of laboratory materials that prevents unnecessary storage or shortage
50. The laboratory has standard operating procedures (Rejection, transport, retention and disposal) and follows it properly
51. The laboratory work environment is organized and clean at all times that specimen handling mechanisms ensures safety for the service providers and users
52. The laboratory has a health and safety manual with procedures that include different types of actions (handling fire and chemical hazard etc.)
53. The laboratory management prepares an established policy for data and information management on data safety, confidentiality, data storing period and disposal mechanisms
54. Does the laboratory have and implements a quality assurance policy that covers all aspects of laboratory functions
**CHAPTER 7. INFECTION PREVENTION AND PATIENT SAFETY**
55. Infection prevention and patient safety committee is established
56. Infection prevention and patient safety committee implementation guideline is prepared
57. Infection prevention and patient safety committee has prepared action plan
58. Infection prevention and patient safety committee plan vs performance report is reviewed by the health center management body
59. Major communicable diseases at the health centers are identified
60. Standard practices to prevent, control and reduce risk of HCAIs are in place and transmission based precautions (TBP) are adequately addressed
61. Health center infection prevention activity monitoring and implementation is based on routes of infection transmission
62. The Health center ensures that equipment, supplies and facilities/infrastructure necessary for infection prevention are available.
63. In order to implement Infection prevention standard and patient safety activities, essential antiseptic and disinfectants are fulfilled in sufficient and appropriate composition
64. All health center staff are trained using standard infection prevention training materials.
65. The Health center provides health education to patients, caregivers and visitors, as appropriate on infection prevention practices
66. Infection prevention and patient safety activities at the health center are also harmonized with the satellite health post and health development groups activity at community level
**CHAPTER 8. MEDICAL EQUIPMENT MANAGEMENT**
67. The health center conducts annual inventor and has a paper-based or computer-based inventory management system that tracks all equipment included in the equipment management program.
68. Health center assigned head of medical equipment technicians accountable to the head of the health center
69. All new equipment undergoes acceptance testing prior to its initial use to ensure the equipment is in good operating condition. Equipment is installed and commissioned in accordance with the manufacturer's specifications.
70. All equipment users are appropriately trained on the operation and maintenance of medical equipment with standard operating procedures readily available to the user. All equipment users are trained on emergency and fire accident prevention and control techniques
71. The health center owns a building plan that is registered by its name, fenced compound, well-functioning waste disposal system, toilet and clean and safe and green recreational/garden areas

72. There is medical equipment and facility engineering administration policy manual /guidance at health center indicating the health center receives Medical equipment maintenance technical support from selected joint coordination bodies such as hospital, regional health bureau or their outsourced institution based on contractual or MOU agreement
73. For health center medical engineering administration there is a medical equipment maintenance request, opening, closing and reporting formats readily available
74. The health center established system on use of facility cars with formal entry and exit control at the facility gates
75. For health center essential health service care, there is 24/7 water and electric power supply or its alternatives
76. Health center has an advance emergency preparedness and response plan on fire and emergency conditions
**CHAPTER 9. HUMAN RESOURCE MANAGEMENT**
77. Personnel are assigned to provide Human Resource Management services.
78. Each employee has a personnel file that is maintained by the HRM case team or case worker.
79. A Human Resource Development plan has been developed
80. Job descriptions have been adopted and documented for each position at the health center.
81. Performance evaluation has been made every 6 month for each employee.
82. Employee benefits and motivation package developed and implemented.
83. Standardized uniforms and badges are worn by staff.
**CHAPTER 10. PERFORMANCE MONITORING AND QUALITY IMPROVEMENT**
84. The health center should establish a structure (multidisciplinary team) for performance monitoring and quality improvement
85. The health center needs to prepare an annual and quarterly plan
86. The health center needs to implement quality improvement cycle for selected priority problems
87. The health center needs to collect, analyze and use for quality improvement purposes and report reportable indicators to the respective body as per the HMIS standard
88. Client satisfaction survey and other quality improvement assessments are conducted, analyzed and applied for quality improvement purposes
**CHAPTER 11. FINANCIAL AND ASSET MANAGEMENT**
89. Bilingual fee posters are displayed beside each departmental reception desk, in all waiting areas and at all cash points. Each poster shows the fees and exemptions and advises patients to obtain and keep receipts for all payments.
90. The health center Accountant prepares a monthly report for the Health Center Management with details of credit granted, credit repaid and balance outstanding.
91. Monthly reconciliation is undertaken for every health center bank account and any donor grants.
92. Monthly and quarterly reports on revenue, expenditures, receivables, payables, trial balance, the status of budget utilization and others including the health center's operating margin are prepared by the Finance Department and submitted to the health center management and Governing Body.
93. Internal and external audit of health center accounts is conducted as a minimum annually and audit reports are reviewed by the Governing Body.
94. The health center has a Memorandum of Understanding with Waiver Certificate Granting Authorities providing details on the type of service and mode of payment.
95. In cases where non-clinical services are outsourced, procedures are in place to monitor the contract and services provided.

96.	There is a current health center accounting manual which establishes all policies and procedures relating to financial management in the hospital.
97.	The health center has a procurement policy, approved by the Senior Management Team that details: The process of submitting procurement requests
98.	There is responsible body/person for approval of procurement requests
99.	Responsible person(s) for purchasing activities
